# Endothelin-1 Signaling Mediates Hypoxia-Induced Microglial Activation Through Reactive Oxygen Species and Mitogen-Activated Protein Kinase Pathways

**DOI:** 10.1080/17590914.2026.2698262

**Published:** 2026-08-05

**Authors:** Yandy Garcia, Ricardo Vázquez, Yalimar P. Pomales-Inostroza, Sebastian Castaño, Francisco Rodriguez, Blake Bailey, Yaritza Inostroza-Nieves

**Affiliations:** a Department of Biochemistry and Pharmacology, San Juan Bautista School of Medicine, Caguas, PR, USA; b Department of Microbiology, University of Puerto Rico, Humacao, PR, USA

**Keywords:** Endothelin-1, hypoxia, interleukin-6, microglia, mitogen-activated protein kinase signaling, neuroinflammation, reactive oxygen species

## Abstract

Oxidative stress and neuroinflammation are critical contributors to hypoxic-ischemic brain injury. Microglia, the CNS-resident immune cells, undergo rapid activation in response to hypoxic stress. Endothelin-1 (ET-1), a vasoconstrictor implicated in cerebrovascular pathology, is upregulated by hypoxia; however, its role in microglial activation remains poorly understood. HMC3 human microglial cells were exposed to hypoxia (1% O_2_) for 4 hours. Reactive oxygen species (ROS) were quantified by flow cytometry. ET-1 and interleukin-6 (IL-6) protein concentrations were measured by ELISA and mRNA levels by qPCR. Mitogen-activated protein kinase (MAPK) activation and ET-1 localization were assessed by flow cytometry and immunofluorescence, respectively. The endothelin B receptor (ET_B_R) antagonist BQ788 was used to assess ET-1 signaling in hypoxia-induced responses. Hypoxia significantly upregulated ET-1 gene expression (5.0-fold increase, *p* < 0.001, *n* = 4) and elevated ET-1 protein production by 1.4-fold (*p* < 0.01, *n* = 4). IL-6 expression and secretion increased 1.5-fold under hypoxic conditions (*p* < 0.01, *n* = 4), an effect that was attenuated by BQ788 pretreatment. ROS levels increased 1.9-fold in hypoxic HMC3 cells (*p* < 0.01, *n* = 4) but were significantly reduced by ET_B_R inhibition. Additionally, hypoxia elevated the percentage of MAPK-activated cells compared to both normoxic and BQ788-treated groups (*p* < 0.01). These findings demonstrate that hypoxia induces ET-1 overexpression, ROS generation, MAPK activation, and IL-6 production in microglia, establishing a self-perpetuating cycle of neuroinflammation. ET_B_R blockade with BQ788 disrupts this cascade, suggesting that ET-1 signaling is a promising therapeutic target to mitigate secondary injury in hypoxic-ischemic brain conditions.

## Introduction

Hypoxic-ischemic (HI) brain injury represents a leading cause of neurological disability and mortality worldwide, occurring when cerebral tissue experiences prolonged oxygen or blood flow deprivation (Dirnagl et al., 1999; Moskowitz et al., 2010). This pathological condition initiates a complex cascade of biochemical and cellular events that ultimately result in neuronal death and central nervous system (CNS) dysfunction. Multiple etiological factors contribute to HI injury, including cerebral hypoxia, atherothrombotic and microangiopathic processes, ischemic or hemorrhagic events, metabolic disturbances, and systemic hypotension (Dirnagl et al., 1999; Moskowitz et al., 2010). The pathophysiological mechanisms underlying HI injury are multifaceted, involving excitotoxicity, oxidative stress, inflammation, and apoptosis (Dirnagl et al., 1999; Moskowitz et al., 2010).

Microglia, the resident immune cells of the CNS, play a pivotal role in the neuroinflammatory response to HI injury (Colonna & Butovsky, 2017; Hanisch & Kettenmann, 2007). Under physiological conditions, microglia maintain CNS homeostasis through immune surveillance, synaptic pruning, and debris clearance (Colonna & Butovsky, 2017). However, in response to pathological stimuli such as hypoxia, microglia undergo rapid morphological and functional transformation, transitioning from a ramified, surveillant phenotype to an activated, amoeboid state (Colonna & Butovsky, 2017; Hanisch & Kettenmann, 2007). Activated microglia produce a diverse array of pro-inflammatory mediators, including cytokines (tumor necrosis factor-alpha [TNF-α], interleukin-1 beta [IL-1β], interleukin-6 [IL-6]), chemokines, reactive oxygen species (ROS), and nitric oxide (NO), which collectively contribute to secondary neuronal injury (Colonna & Butovsky, 2017; Hanisch & Kettenmann, 2007).

Endothelin-1 (ET-1) is a 21-amino acid vasoactive peptide originally identified for its potent vasoconstrictive properties (Yanagisawa et al., 1988). ET-1 exerts its biological effects through two G-protein-coupled receptors: endothelin A receptor (ET_A_R) and endothelin B receptor (ET_B_R) (Davenport et al., 2016). While ET_A_R is predominantly expressed on vascular smooth muscle cells and mediates vasoconstriction, ET_B_R is found on endothelial cells, where it promotes vasodilation through nitric oxide release, and on various other cell types, including astrocytes and microglia, where it can mediate pro-inflammatory responses (Davenport et al., 2016; Koyama et al., 2013). Accumulating evidence implicates ET-1 in the pathogenesis of various neurological disorders, including stroke, traumatic brain injury, subarachnoid hemorrhage, and neurodegenerative diseases (Briyal et al., 2015; Schinelli, 2006). ET-1 levels are elevated in the cerebrospinal fluid and brain tissue following ischemic stroke, and exogenous ET-1 administration exacerbates ischemic brain injury in experimental models (Briyal et al., 2015; Schinelli, 2006).

Hypoxia is a potent stimulus for ET-1 production in various cell types, including endothelial cells, cardiomyocytes, and neurons (Elton et al., 1992; Yamashita et al., 2001). Hypoxia-inducible factor-1 alpha (HIF-1α), a master transcriptional regulator of cellular responses to hypoxia, directly binds to the ET-1 gene promoter and enhances its transcription (Yamashita et al., 2001). However, the specific role of ET-1 in hypoxia-induced microglial activation remains poorly characterized. Given that microglia are key mediators of neuroinflammation in HI injury and that ET-1 is upregulated under hypoxic conditions, we hypothesized that ET-1 contributes to hypoxia-induced microglial activation through ET_B_R-mediated signaling pathways.

The present study investigated the effects of hypoxia on ET-1 expression, ROS production, inflammatory cytokine release, and MAPK activation in the human microglial cell line HMC3. We further examined the role of ET_B_R in mediating these hypoxia-induced responses using the selective ET_B_R antagonist BQ788. Our findings provide novel insights into the molecular mechanisms linking hypoxia, ET-1 signaling, and microglial activation, with potential implications for therapeutic intervention in HI brain injury.

## Materials and Methods

### Cell Culture and Hypoxia Exposure

The human microglial cell line HMC3 (ATCC CRL-3304) was cultured in Eagle’s Minimum Essential Medium (EMEM; ATCC 30–2003) supplemented with 10% fetal bovine serum (FBS; Gibco) and 1% penicillin-streptomycin (Gibco) at 37 °C in a humidified atmosphere containing 5% CO_2_. Cells were seeded at a density of 1 × 10^5^ cells/well in 6-well plates and allowed to adhere overnight. For hypoxia experiments, cells were placed in a modular incubator chamber (Billups-Rothenberg) flushed with a gas mixture of 1% O_2_, 5% CO_2_, and 94% N_2_ for 10 minutes to establish hypoxic conditions. The chamber was sealed and incubated at 37 °C for 4 hours. Normoxic control cells were maintained under standard culture conditions (21% O_2_, 5% CO_2_) for the same duration. The 4-hour exposure represents a transient hypoxic insult; following hypoxia, cells were returned to standard normoxic conditions (reoxygenation) to model the hypoxia-reoxygenation injury characteristic of hypoxic-ischemic events. Cells and conditioned media were harvested for analysis at 0, 24, and 48 hours after the end of the hypoxic period, with matched normoxic controls processed in parallel at each time point. Unless otherwise indicated, the values reported in the Results correspond to the time point at which the maximal response was observed for each readout.

### Pharmacological Treatments

To investigate the role of ET_B_R in hypoxia-induced microglial responses, cells were pretreated with the selective ET_B_R antagonist BQ788 (Tocris Bioscience; 1 μM) for 30 minutes prior to hypoxia exposure. BQ788 was dissolved in dimethyl sulfoxide (DMSO; Sigma-Aldrich) at a stock concentration of 10 mM and diluted in culture medium to the working concentration. The final DMSO concentration in culture medium did not exceed 0.01% (v/v). Vehicle control cells received an equivalent volume of DMSO. The 1 μM concentration was selected based on the high selectivity and sub-nanomolar to nanomolar affinity of BQ788 for the ETBR, which provides effective receptor blockade at the micromolar range while minimizing off-target effects on the ETAR, and is consistent with the concentration used to inhibit ET-1/ETBR signaling in HMC3 microglia in a previous work (Inostroza-Nieves et al., 2025). BQ788 was added 30 minutes before hypoxia and was maintained in the culture medium throughout the hypoxic exposure and the subsequent reoxygenation period (it was not washed out). This pre-incubation ensured that the ETBR was blocked before hypoxia-induced ET-1 production, while the continued presence of the antagonist ensured sustained receptor blockade throughout the entire response period.

### Quantitative Real-Time PCR (qPCR)

Total RNA was extracted from HMC3 cells using TRIzol reagent (Invitrogen, Carlsbad, CA, USA) according to the manufacturer’s protocol. RNA concentration and purity were assessed using a NanoDrop spectrophotometer (Thermo Fisher Scientific, Waltham, MA, USA). First-strand complementary DNA (cDNA) was synthesized from 1 μg of total RNA using the High Capacity cDNA Reverse Transcription Kit (Applied Biosystems, Foster City, CA, USA). Quantitative PCR was performed using TaqMan^™^ Gene Expression Master Mix (Applied Biosystems, Foster City, CA, USA) on a StepOnePlus Real-Time PCR System (Applied Biosystems). TaqMan gene expression assay for EDN (Hs00174961_m1), IL6 (Hs00174131_m1), and GAPDH (Hs99999905_m1) (Applied Biosystems) were used. Thermal cycling conditions consisted of an initial denaturation at 95 °C for 10 minutes, followed by 40 cycles of 95 °C for 15 seconds and 60 °C for 1 minute. Relative gene expression was calculated using the 2^−ΔΔCt^ method, with glyceraldehyde 3-phosphate dehydrogenase (GAPDH) serving as the internal reference gene. Each sample was analyzed in triplicate, and data are presented as fold change relative to normoxic controls.

### Enzyme-Linked Immunosorbent Assay (ELISA)

Culture supernatants were collected following hypoxia exposure and centrifuged at 1,000 × *g* for 5 minutes to remove cellular debris. ET-1 and IL-6 protein concentrations were quantified using commercially available ELISA kits (R&D Systems) according to the manufacturer’s protocols. Absorbance was measured at 450 nm using a microplate reader (Thermo Scientific), with wavelength correction set at 540 nm. Protein concentrations were determined by interpolation from standard curves generated using recombinant human ET-1 and IL-6 standards.

### Reactive Oxygen Species (ROS) Detection

Intracellular ROS levels were assessed using the Muse Oxidative Stress Kit (Luminex Corporation) according to the manufacturer’s instructions. Briefly, HMC3 cells were harvested by trypsinization, washed with phosphate-buffered saline (PBS), and resuspended in culture medium at a concentration of 5 × 10^5^ cells/mL. Cells were incubated with dihydroethidium (DHE) working solution for 30 minutes at 37 °C in the dark. Following incubation, cells were analyzed using the Muse Cell Analyzer (Luminex Corporation). The percentage of ROS-positive cells was determined by flow cytometry, with gating based on fluorescence intensity. Forward- and side-scatter gating was applied to exclude debris and non-viable cells, so that all reported flow cytometry measurements (ROS and MAPK) were derived from the viable cell population. Under the hypoxia-reoxygenation conditions used here, cell viability remained high (>90% by trypan blue exclusion), and no overt cytotoxicity was observed, indicating that the responses measured reflect signaling in surviving microglia rather than cell death.

### MAPK Activation Assay

MAPK activation was evaluated using the Muse MAPK Activation Dual Detection Kit (Luminex Corporation), which simultaneously detects phosphorylated and total levels of extracellular signal-regulated kinase 1/2 (ERK1/2) and p38 MAPK. HMC3 cells were harvested, fixed, and permeabilized according to the manufacturer’s protocol. Cells were then incubated with fluorescently labeled antibodies specific for phospho-ERK1/2 (Thr202/Tyr204) and phospho-p38 MAPK (Thr180/Tyr182), as well as antibodies recognizing total ERK1/2 and p38 MAPK. Following antibody incubation, cells were analyzed using the Muse Cell Analyzer. The percentage of cells exhibiting MAPK activation was determined based on the ratio of phosphorylated to total MAPK protein levels.

### Immunofluorescence Microscopy

HMC3 cells were seeded on glass coverslips in 24-well plates and subjected to normoxic or hypoxic conditions as described above. Following treatment, cells were fixed with 4% paraformaldehyde in PBS for 15 minutes at room temperature, permeabilized with 0.1% Triton X-100 in PBS for 10 minutes, and blocked with 5% bovine serum albumin (BSA) in PBS for 1 hour. Cells were then incubated overnight at 4 °C with a rabbit polyclonal anti-ET-1 antibody (Abcam; 1:200 dilution) in blocking buffer. After washing with PBS, cells were incubated with Alexa Fluor 488-conjugated goat anti-rabbit IgG secondary antibody (Invitrogen; 1:500 dilution) for 1 hour at room temperature in the dark. Nuclei were counterstained with 4′,6-diamidino-2-phenylindole (DAPI; Sigma-Aldrich; 1 μg/mL) for 5 minutes. Coverslips were mounted on glass slides using ProLong Gold Antifade Mountant (Invitrogen). Fluorescence images were captured using a Nikon Eclipse Ti-E Inverted Fluorescence Microscope at 10× magnification.

### Statistical Analysis

All experiments were performed in four independent biological replicates (n = 4), where each biological replicate represents a separate, independently treated culture derived from a distinct cell passage; qPCR measurements were additionally performed in technical triplicate within each biological replicate. Data are presented as mean ± standard deviation (SD). Statistical comparisons between two groups were performed using unpaired Student’s *t*-tests. Multiple group comparisons were analyzed by one-way analysis of variance (ANOVA) followed by Tukey’s *post hoc* test for pairwise comparisons. Statistical significance was defined as *p* < 0.05. All statistical analyses were conducted using GraphPad Prism version 9.0 (GraphPad Software).

## Results

### Hypoxia Upregulates ET-1 Expression in HMC3 Microglial Cells

**Figure 1.**
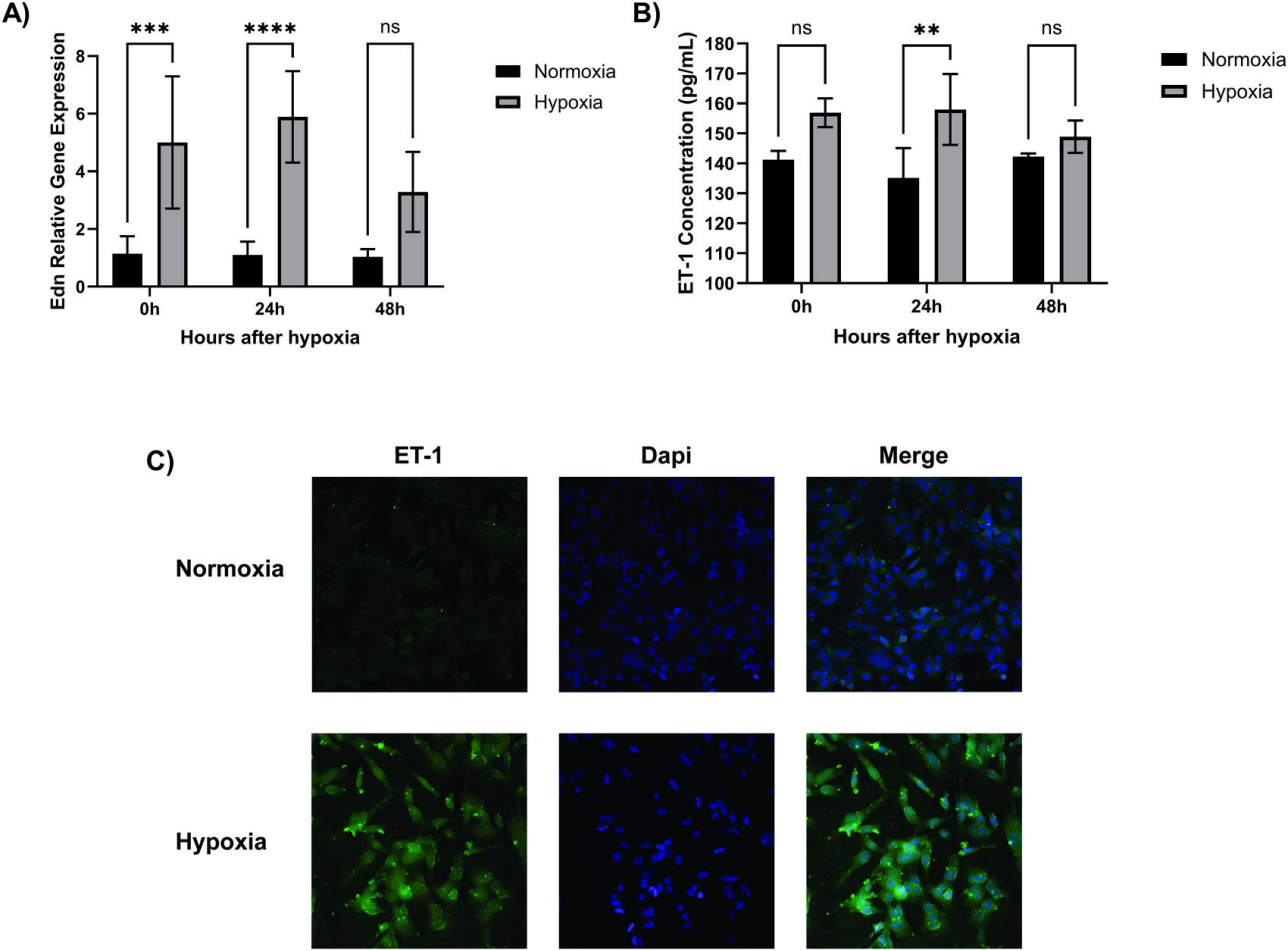
Hypoxia increases ET-1 gene expression and protein concentration in human microglial cells. (A) Quantitative analysis of ET-1 mRNA levels in HMC3 cells cultured under normoxic and hypoxic conditions at 0, 24, and 48 hours after hypoxia. (B) ET-1 protein concentration (pg/mL) in the culture media at corresponding time points, measured by ELISA. A significant increase was observed at 24 h after hypoxia compared to normoxia. (C) Representative fluorescence microscopy images showing ET-1 immunoreactivity (green) and DAPI-stained nuclei (blue) under normoxia and hypoxia. Merged panels demonstrate enhanced ET-1 localization and intensity under hypoxic conditions. Data are expressed as fold change relative to normoxia (mean ± SD, n = 4). Statistical significance: *p < 0.05, **p < 0.01, ***p < 0.001.

To investigate whether hypoxia induces ET-1 expression in microglia, HMC3 cells were exposed to hypoxic conditions (1% O_2_) for 4 hours, followed by reoxygenation under normoxic conditions, and ET-1 mRNA and protein levels were assessed at 0, 24, and 48 hours after hypoxia. Quantitative real-time PCR analysis revealed that ET-1 mRNA expression peaked at 24 hours after hypoxia, with a significant 5.0-fold increase relative to normoxic controls (*p* < 0.001, *n* = 4; Figure 1A). In contrast to the transient mRNA response, ELISA measurements demonstrated a 1.4-fold elevation in secreted ET-1 protein in the culture medium that remained significantly elevated at 24 and 48 hours after hypoxia, consistent with the accumulation and longer half-life of the secreted peptide relative to its transcript (*p* < 0.01, *n* = 4; Figure 1B). Immunofluorescence microscopy confirmed increased ET-1 protein expression in hypoxic HMC3 cells, with enhanced cytoplasmic and perinuclear staining compared to normoxic controls (Figure 1C). Because HMC3 is a clonal, homogeneous human microglial cell line cultured as a monoculture, all DAPI-positive cells in the field are microglia; the ET-1 immunoreactivity therefore reflects intracellular ET-1 within microglial cells. These findings establish that hypoxia is a potent stimulus for ET-1 production in human microglia.

### Hypoxia Induces IL-6 Expression and Secretion in Microglia Via ET_B_R

**Figure 2.**
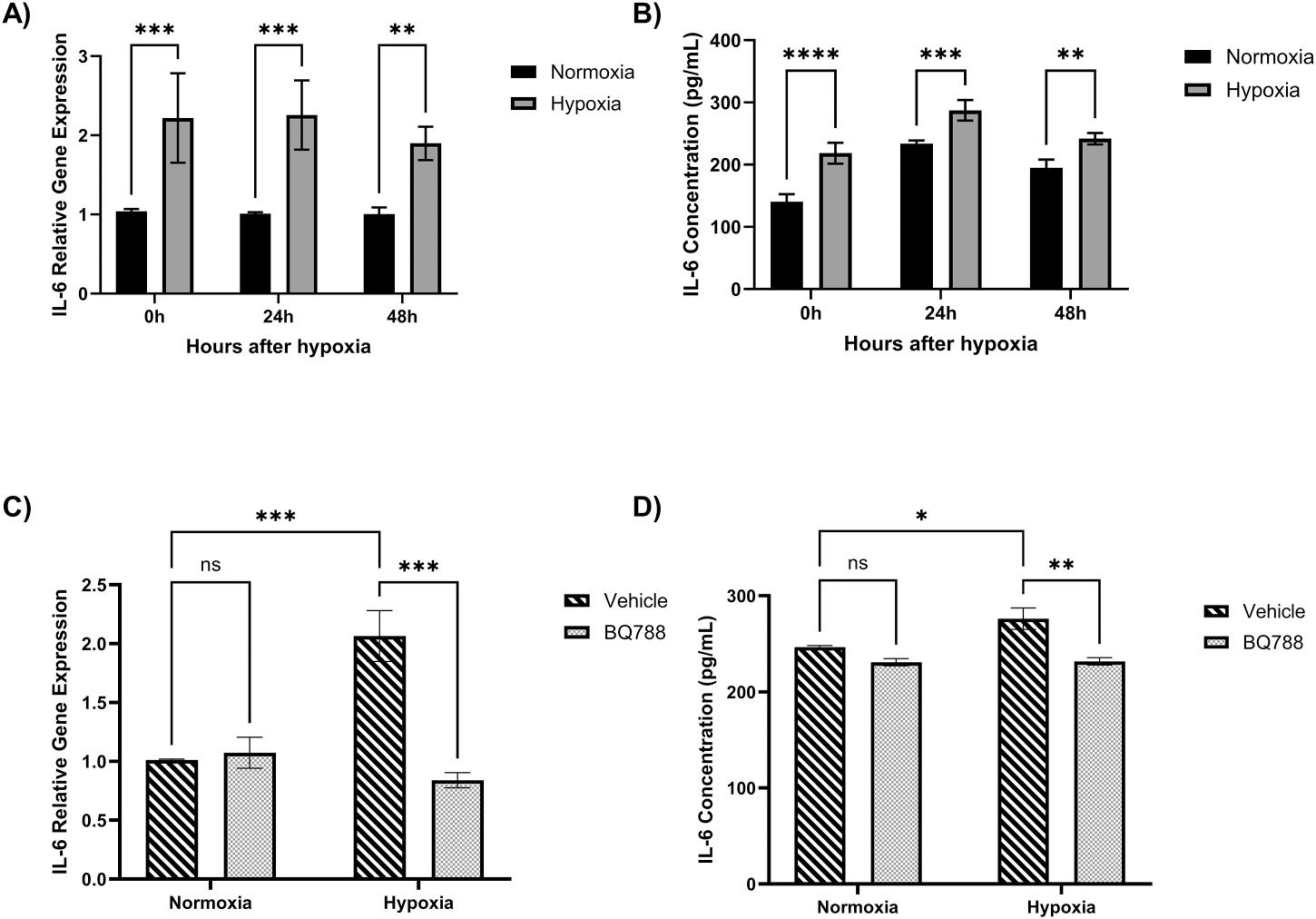
ETBR antagonism reduces hypoxia-induced IL-6 expression and secretion in microglial cells. (A) Relative gene expression of IL-6 right after, 24 hours or 48 hours after hypoxia or normoxia. (B) IL-6 concentration (pg/mL) measured by ELISA under the same conditions. (C) Relative gene expression of IL-6 under normoxia or hypoxia with vehicle or BQ788 (1 μM) treatment. (D) IL-6 concentration (pg/mL) measured by ELISA under the same conditions as C. Under hypoxia, IL-6 expression and secretion were significantly increased in vehicle-treated cells and reduced by BQ788 treatment (*p* < 0.05, n = 4). No significant changes were observed under normoxia. Data represent mean ± SD.

Given that IL-6 is a key pro-inflammatory cytokine produced by activated microglia, we examined whether hypoxia modulates IL-6 expression in HMC3 cells. Hypoxia exposure resulted in a 1.5-fold increase in IL-6 mRNA expression, measured by qPCR in the cell lysate, (*p* < 0.01, *n* = 4; Figure 2A) and a corresponding 1.5-fold elevation in secreted IL-6 protein, measured by ELISA in the conditioned culture medium (*p* < 0.01, *n* = 4; Figure 2B). These results demonstrate that hypoxia triggers a pro-inflammatory response in microglia characterized by increased IL-6 production.

To determine whether ET-1 signaling contributes to hypoxia-induced IL-6 production, HMC3 cells were pretreated with the selective ET_B_R antagonist BQ788 (1 μM) prior to hypoxia exposure. BQ788 pretreatment significantly reduced hypoxia-induced IL-6 mRNA expression (Figure 2C) and protein secretion (Figure 2D) to levels comparable to normoxic controls (*p* < 0.01, *n* = 4). These findings indicate that ET_B_R signaling plays a critical role in mediating hypoxia-induced IL-6 production in microglia.

### Hypoxia Increases ROS Generation Through ET_B_R-Dependent Mechanisms

**Figure 3.**
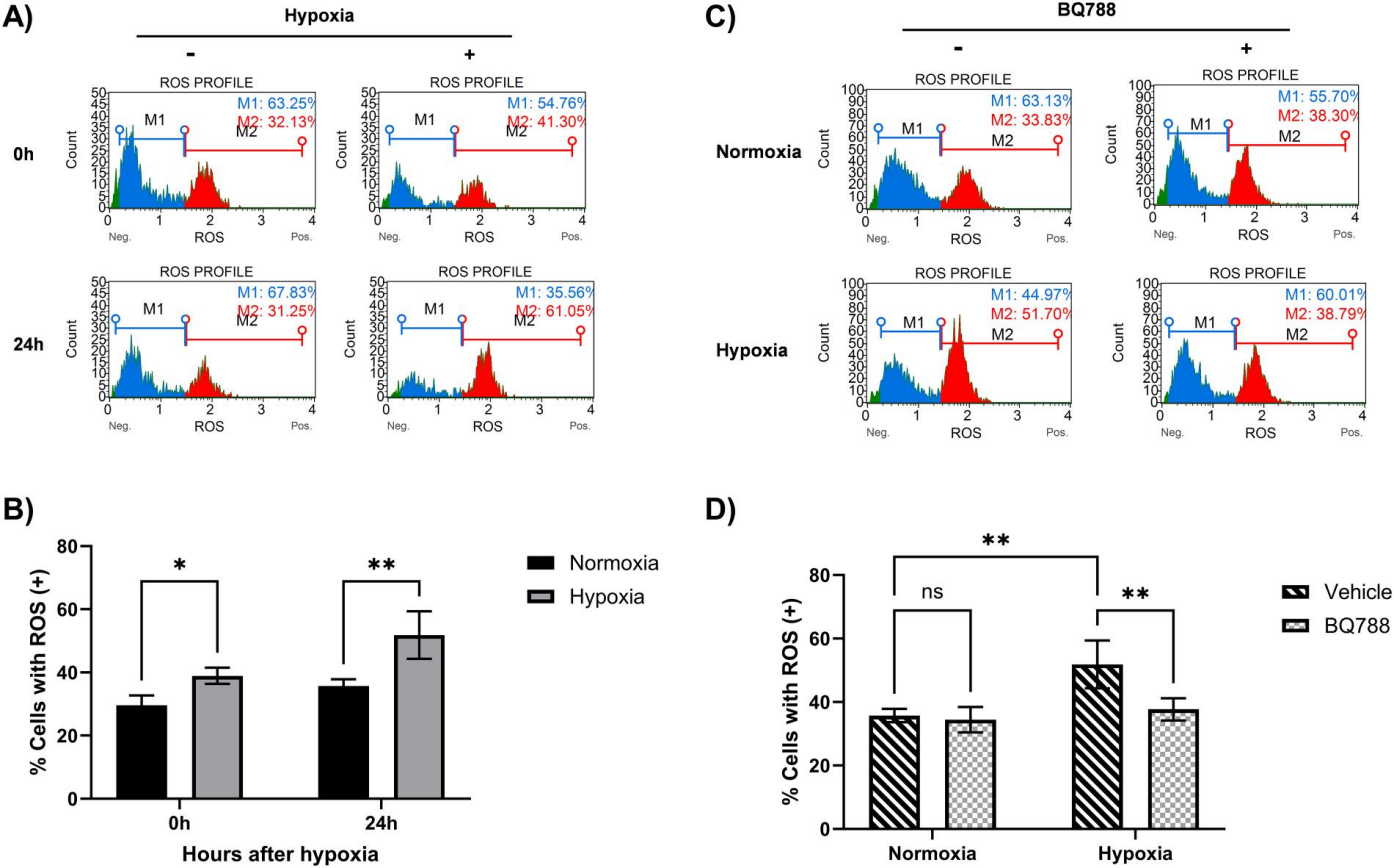
Hypoxia elevates reactive oxygen species (ROS) generation, while ETBR blockade attenuates oxidative stress. (A) Representative flow cytometry histograms showing shifts in ROS fluorescence intensity (M1 = low ROS, M2 = high ROS) in HMC3 cells under normoxia and hypoxia (0 h and 24 h after hypoxia). (B) Quantification of ROS-positive cells after hypoxia exposure, showing a significant increase in ROS production at just after and 24 h after hypoxia compared to normoxia (*p < 0.01). (C–D) Effect of BQ788 (1 μM) treatment on ROS generation under normoxic and hypoxic conditions. Hypoxia-induced ROS accumulation was significantly reduced by BQ788 treatment (*p* < 0.05). Data are expressed as mean ± SD, n = 4.

Oxidative stress is a hallmark of hypoxic injury and a key mediator of microglial activation. Flow cytometric analysis using the Muse Oxidative Stress Assay revealed a 1.9-fold increase in the percentage of ROS-positive HMC3 cells following hypoxia exposure (*p* < 0.01, *n* = 4; Figure 3A). Notably, pretreatment with BQ788 significantly attenuated hypoxia-induced ROS generation, reducing ROS-positive cells to levels not significantly different from normoxic controls (Figure 3A). Representative flow cytometry histograms illustrating the shift in ROS fluorescence intensity are shown in Figure 3B. These results demonstrate that ET_B_R signaling is required for hypoxia-induced ROS production in microglia.

### Hypoxia Activates MAPK Signaling Pathways in Microglia

**Figure 4.**
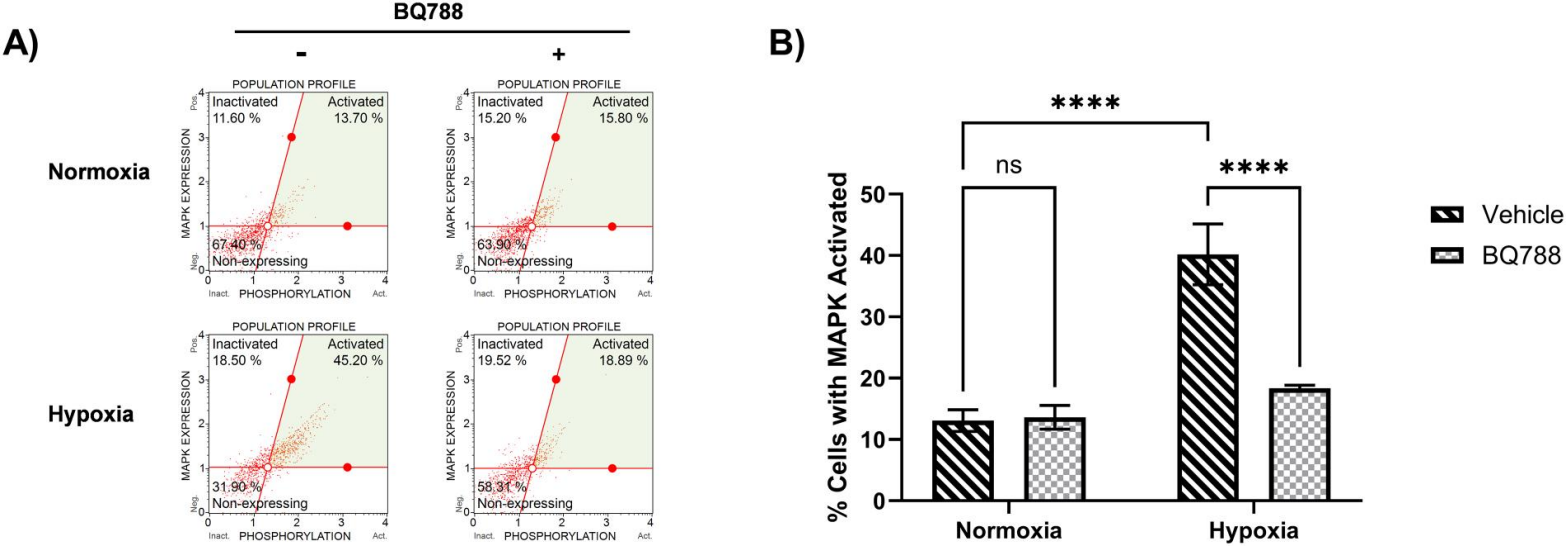
ETBR inhibition suppresses MAPK activation in hypoxic microglial cells. (A) Representative flow cytometry scatter plots showing the distribution of MAPK expression and phosphorylation states under normoxic and hypoxic conditions, with and without BQ788 treatment. Cell populations are identified as non-expressing, inactivated, or activated MAPK-positive cells. (B) Quantitative analysis of MAPK activation (% of total cells). Hypoxia significantly increased MAPK activation compared to normoxia, while BQ788 treatment markedly reduced the percentage of activated cells (*p < 0.01). Data represent mean ± SD, n = 4.

Mitogen-activated protein kinases (MAPKs), including ERK1/2 and p38 MAPK, are critical signaling molecules that regulate microglial activation and inflammatory responses. Using the Muse MAPK Activation Dual Detection Kit, we assessed the phosphorylation status of ERK1/2 and p38 MAPK in HMC3 cells following hypoxia exposure. Hypoxia significantly increased the percentage of cells exhibiting MAPK activation compared to normoxic controls (*p* < 0.01, *n* = 4; Figure 4A). Pretreatment with BQ788 attenuated hypoxia-induced MAPK activation, reducing the percentage of MAPK-activated cells to levels comparable to normoxic controls (Figure 4A). Representative flow cytometry dot plots illustrating MAPK activation profiles are shown in Figure 4B. These findings indicate that ET_B_R signaling is upstream of MAPK activation in hypoxia-induced microglial responses.

## Discussion

The present study provides novel evidence that hypoxia induces ET-1 overexpression in human microglia and that ET-1 signaling through ET_B_R mediates hypoxia-induced ROS generation, IL-6 production, and MAPK activation. These findings establish a mechanistic link between hypoxia, ET-1 signaling, and microglial activation, with important implications for understanding the pathophysiology of hypoxic-ischemic brain injury.

### Hypoxia-Induced ET-1 Expression in Microglia

Our observation that hypoxia upregulates ET-1 expression in HMC3 microglial cells is consistent with previous reports demonstrating hypoxia-induced ET-1 production in various cell types, including endothelial cells, cardiomyocytes, and neurons (Elton et al., 1992; Yamashita et al., 2001). The transcriptional upregulation of ET-1 under hypoxic conditions is primarily mediated by HIF-1α, which binds to hypoxia-response elements in the ET-1 gene promoter (Yamashita et al., 2001). While HIF-1α-dependent ET-1 induction has been well characterized in endothelial cells, our findings extend this mechanism to microglia, suggesting that microglia may serve as an additional cellular source of ET-1 in the hypoxic brain. The local production of ET-1 by activated microglia could contribute to both autocrine and paracrine signaling, amplifying neuroinflammatory responses and exacerbating tissue injury.

### ET_B_R-Mediated IL-6 Production

Interleukin-6 is a pleiotropic cytokine that plays complex roles in neuroinflammation, exhibiting both pro-inflammatory and anti-inflammatory properties depending on the cellular context and signaling pathways involved (Rothaug et al., 2016). In the context of acute brain injury, elevated IL-6 levels are generally associated with adverse outcomes, contributing to blood-brain barrier disruption, neuronal apoptosis, and glial scar formation (Rothaug et al., 2016). Our finding that hypoxia induces IL-6 expression and secretion in microglia is consistent with previous studies demonstrating hypoxia-induced IL-6 production in various cell types (Ambler et al., 2012; Yamauchi-Takihara et al., 1995). Importantly, we demonstrate that ET_B_R antagonism with BQ788 significantly attenuates hypoxia-induced IL-6 production, establishing ET-1/ET_B_R signaling as an upstream regulator of IL-6 expression in hypoxic microglia. This finding is consistent with previous reports showing that ET-1 stimulates IL-6 production in astrocytes through ET_B_R-dependent mechanisms (Koyama et al., 2013).

### ET_B_R-Dependent ROS Generation

Reactive oxygen species play a dual role in cellular physiology, serving as important signaling molecules at physiological concentrations but causing oxidative damage to lipids, proteins, and nucleic acids when produced in excess (Schieber & Chandel, 2014). In the context of hypoxic-ischemic brain injury, excessive ROS production contributes to neuronal death, blood-brain barrier disruption, and inflammatory responses (Schieber & Chandel, 2014). Our observation that hypoxia increases ROS generation in microglia and that this effect is attenuated by ET_B_R antagonism suggests that ET-1 signaling is a critical regulator of oxidative stress in hypoxic microglia. The mechanism by which ET_B_R activation stimulates ROS production likely involves activation of NADPH oxidase, a multi-subunit enzyme complex that catalyzes the production of superoxide anion (Romero et al., 2009). Previous studies have demonstrated that ET-1 stimulates NADPH oxidase-dependent ROS production in vascular smooth muscle cells and endothelial cells (Romero et al., 2009), and our findings extend this mechanism to microglia.

### MAPK Activation in Hypoxic Microglia

Mitogen-activated protein kinases are evolutionarily conserved serine/threonine kinases that regulate diverse cellular processes, including proliferation, differentiation, apoptosis, and inflammatory responses (Cargnello & Roux, 2011). In microglia, MAPK signaling pathways play critical roles in regulating the production of pro-inflammatory mediators and the transition between different activation states (Kaminska et al., 2016). Our finding that hypoxia activates MAPK signaling in HMC3 cells and that this activation is attenuated by ET_B_R antagonism suggests that ET-1/ET_B_R signaling is upstream of MAPK activation in hypoxic microglia. The activation of MAPK pathways by ET-1 has been reported in various cell types, including vascular smooth muscle cells, cardiomyocytes, and astrocytes (Koyama et al., 2013). ROS can serve as upstream activators of MAPK signaling pathways (Son et al., 2011), suggesting that ET_B_R-mediated ROS generation may contribute to MAPK activation in hypoxic microglia. This hypothesis is supported by our observation that ET_B_R antagonism attenuates both ROS generation and MAPK activation. We note, however, that because both responses were suppressed by the same upstream intervention (ET_B_R blockade), our data establish that ROS generation and MAPK activation are both downstream of ET_B_R signaling but do not, by themselves, demonstrate that ROS is the direct and necessary cause of MAPK activation; this sequential relationship remains correlative in the present study and is inferred from the established literature linking ROS to MAPK signaling (Son et al., 2011). Direct demonstration of this link would require pharmacological ROS scavenging (e.g., N-acetylcysteine or apocynin) to test whether quenching ROS specifically prevents MAPK activation and downstream IL-6 secretion, and we identify this as an important direction for future work.

### Proposed Mechanism: A Self-Perpetuating Cycle of Neuroinflammation

Based on our findings, we propose the following mechanistic model for hypoxia-induced microglial activation: (1) Hypoxia upregulates ET-1 expression in microglia through HIF-1α-dependent transcriptional mechanisms; (2) ET-1 acts in an autocrine manner to activate ET_B_R on microglial cells; (3) ET_B_R activation stimulates ROS generation, likely through NADPH oxidase; (4) ROS activate MAPK signaling pathways; (5) MAPK activation leads to increased transcription and secretion of pro-inflammatory cytokines, including IL-6; (6) IL-6 and other pro-inflammatory mediators further amplify microglial activation and may stimulate additional ET-1 production, establishing a self-perpetuating cycle of neuroinflammation. This model is consistent with the concept of “reactive microgliosis,” in which initial microglial activation triggers a cascade of inflammatory responses that can persist long after the initial insult has resolved (Colonna & Butovsky, 2017).

### Therapeutic Implications

The identification of ET_B_R as a critical mediator of hypoxia-induced microglial activation suggests that ET_B_R antagonism may represent a promising therapeutic strategy for mitigating neuroinflammation in hypoxic-ischemic brain injury. Several ET receptor antagonists have been developed and tested in clinical trials for cardiovascular indications, although their application in neurological disorders remains limited (Dhaun & Webb, 2019). The blood-brain barrier penetration of existing ET receptor antagonists is generally poor, which has hindered their development for CNS indications (Dhaun & Webb, 2019). However, recent advances in drug delivery technologies, including nanoparticle-based systems and receptor-mediated transcytosis, may enable more effective CNS delivery of ET receptor antagonists (Dhaun & Webb, 2019). Alternatively, the development of novel, brain-penetrant ET_B_R antagonists could facilitate the translation of our findings to clinical applications.

### Limitations and Future Directions

Several limitations of the present study should be acknowledged. First, our experiments were conducted using the HMC3 human microglial cell line, which, while widely used as a model system, may not fully recapitulate the phenotypic and functional characteristics of primary human microglia or microglia *in vivo*. Future studies using primary microglial cultures or *in vivo* models of hypoxic-ischemic brain injury will be necessary to validate our findings in more physiologically relevant systems. Second, our study employed a single 4-hour hypoxic insult followed by reoxygenation, with responses assessed at 0, 24, and 48 hours after hypoxia. While this design captures the early reoxygenation dynamics relevant to hypoxic-ischemic injury, the effects of longer or repeated hypoxic exposures, and of time points beyond 48 hours, remain to be characterized. Third, while we have identified ET_B_R as a critical mediator of hypoxia-induced microglial activation, the intracellular signaling pathways downstream of ET_B_R activation require further elucidation. Future studies employing pharmacological inhibitors or genetic approaches to manipulate specific signaling molecules (e.g., NADPH oxidase subunits, specific MAPK isoforms) will be necessary to fully delineate the signaling cascade linking ET_B_R activation to inflammatory mediator production.

Additionally, the present study focused on ET_B_R as the primary endothelin receptor mediating hypoxia-induced microglial activation, based on previous findings from our laboratory demonstrating that ET_A_R antagonism did not significantly affect ET-1-induced inflammatory responses in HMC3 cells (Inostroza-Nieves et al., 2025). However, the relative expression levels and functional roles of ET_A_R and ET_B_R may vary across different microglial activation states, brain regions, and pathological conditions. Future studies examining potential cell-type-specific or context-dependent roles of ET_A_R in microglial responses would be valuable.

Finally, the potential interactions between ET-1 signaling and other pathways implicated in hypoxic-ischemic brain injury, such as adenosine signaling, glutamate excitotoxicity, and complement activation, warrant investigation. Understanding these interactions may reveal additional therapeutic targets and inform the development of combination therapies for hypoxic-ischemic brain injury.

## Conclusion

This study demonstrates that hypoxia induces ET-1 overexpression in human microglia and that ET-1 signaling through ET_B_R mediates hypoxia-induced ROS generation, IL-6 production, and MAPK activation. These findings establish a mechanistic link between hypoxia, ET-1 signaling, and microglial activation, revealing a self-perpetuating cycle of neuroinflammation in hypoxic-ischemic brain injury. ET_B_R antagonism disrupts this inflammatory cascade, suggesting that targeting ET-1 signaling may represent a promising therapeutic strategy for mitigating secondary injury in hypoxic-ischemic brain conditions. Future studies in primary microglial cultures and *in vivo* models will be necessary to validate these findings and to assess the therapeutic potential of ET_B_R antagonism in clinical settings.

## References

[CIT0001] Ambler, D. R., Fletcher, N. M., Diamond, M. P., & Saed, G. M. (2012). Effects of hypoxia on the expression of inflammatory markers IL-6 and TNF-α in human normal peritoneal and adhesion fibroblasts. *Systems Biology in Reproductive Medicine*, *58*(6), 324–329. 10.3109/19396368.2012.713439 23043632

[CIT0002] Briyal, S., Shepard, C., & Gulati, A. (2015). Endothelin receptor type B agonist, IRL-1620, prevents beta amyloid (Aβ) induced oxidative stress and cognitive impairment in normal and diabetic rats. *Pharmacology Biochemistry and Behavior*, *130*, 65–72. 10.1016/j.pbb.2015.01.004 24561065

[CIT0003] Cargnello, M., & Roux, P. P. (2011). Activation and function of the MAPKs and their substrates, the MAPK-activated protein kinases. *Microbiology and Molecular Biology Reviews: MMBR*, *75*(1), 50–83. 10.1128/MMBR.00031-10 21372320 PMC3063353

[CIT0004] Colonna, M., & Butovsky, O. (2017). Microglia function in the central nervous system during health and neurodegeneration. *Annual Review of Immunology*, *35*(1), 441–468. 10.1146/annurev-immunol-051116-052358 PMC8167938 28226226

[CIT0005] Davenport, A. P., Hyndman, K. A., Dhaun, N., Southan, C., Kohan, D. E., Pollock, J. S., Pollock, D. M., Webb, D. J., & Maguire, J. J. (2016). Endothelin. *Pharmacological Reviews*, *68*(2), 357–418. 10.1124/pr.115.011833 26956245 PMC4815360

[CIT0006] Dhaun, N., & Webb, D. J. (2019). Endothelins in cardiovascular biology and therapeutics. *Nature Reviews. Cardiology*, *16*(8), 491–502. 10.1038/s41569-019-0176-3 30867577

[CIT0007] Dirnagl, U., Iadecola, C., & Moskowitz, M. A. (1999). Pathobiology of ischaemic stroke: An integrated view. *Trends in Neurosciences*, *22*(9), 391–397. 10.1016/S0166-2236(99)01401-0 10441299

[CIT0008] Elton, T. S., Oparil, S., Taylor, G. R., Hicks, P. H., Yang, R. H., Jin, H., & Chen, Y. F. (1992). Normobaric hypoxia stimulates endothelin-1 gene expression in the rat. *The American Journal of Physiology*, *263*(6 Pt 2), R1260–R1264. 10.1152/ajpregu.1992.263.6.R1260 1481936

[CIT0009] Hanisch, U. K., & Kettenmann, H. (2007). Microglia: Active sensor and versatile effector cells in the normal and pathologic brain. *Nature Neuroscience*, *10*(11), 1387–1394. 10.1038/nn1997 17965659

[CIT0010] Inostroza-Nieves, Y., Bou, S., Alvarado, J., Capo-Ruiz, D., Garcia, J., Moliere, J. P., & Arenas, C. P. (2025). Endothelin-1 triggers oxidative stress and cytokine release in human microglia cells through ETRB-dependent mechanisms. *Frontiers in Cellular Neuroscience*, *19*, 1677457. 10.3389/fncel.2025.1677457 41063975 PMC12501885

[CIT0011] Kaminska, B., Mota, M., & Pizzi, M. (2016). Signal transduction and epigenetic mechanisms in the control of microglia activation during neuroinflammation. *Biochimica et Biophysica Acta*, *1862*(3), 339–351. 10.1016/j.bbadis.2015.10.026 26524636

[CIT0012] Koyama, Y., Kotani, M., Sawamura, T., Kuribayashi, M., Konishi, R., & Michinaga, S. (2013). Different actions of endothelin-1 on chemokine production in rat cultured astrocytes. *Journal of Neuroinflammation*, *10*(1), 51. 10.1186/1742-2094-10-51 23627909 PMC3675376

[CIT0013] Moskowitz, M. A., Lo, E. H., & Iadecola, C. (2010). The science of stroke: Mechanisms in search of treatments. *Neuron*, *67*(2), 181–198. 10.1016/j.neuron.2010.07.002 20670828 PMC2957363

[CIT0014] Romero, M., Jiménez, R., Sánchez, M., López-Sepúlveda, R., Zarzuelo, M. J., O’Valle, F., Zarzuelo, A., Pérez-Vizcaíno, F., & Duarte, J. (2009). Quercetin inhibits vascular superoxide production induced by endothelin-1: Role of NADPH oxidase, uncoupled eNOS and PKC. *Atherosclerosis*, *202*(1), 58–67. 10.1016/j.atherosclerosis.2008.03.007 18436224

[CIT0015] Rothaug, M., Becker-Pauly, C., & Rose-John, S. (2016). The role of interleukin-6 signaling in nervous tissue. *Biochimica et Biophysica Acta*, *1863*(6 Pt A), 1218–1227. 10.1016/j.bbamcr.2016.03.018 27016501

[CIT0016] Schieber, M., & Chandel, N. S. (2014). ROS function in redox signaling and oxidative stress. *Current Biology: CB*, *24*(10), R453–R462. 10.1016/j.cub.2014.03.034 24845678 PMC4055301

[CIT0017] Schinelli, S. (2006). Pharmacology and physiopathology of the brain endothelin system: An overview. *Current Medicinal Chemistry*, *13*(6), 627–638. 10.2174/092986706776055652 16529555

[CIT0018] Son, Y., Cheong, Y. K., Kim, N. H., Chung, H. T., Kang, D. G., & Pae, H. O. (2011). Mitogen-activated protein kinases and reactive oxygen species: How can ROS activate MAPK pathways? *Journal of Signal Transduction*, *2011*, 792639–792636. 10.1155/2011/792639 21637379 PMC3100083

[CIT0019] Yamashita, K., Discher, D. J., Hu, J., Bishopric, N. H., & Webster, K. A. (2001). Molecular regulation of the endothelin-1 gene by hypoxia. Contributions of hypoxia-inducible factor-1, activator protein-1, GATA-2, and p300/CBP. *The Journal of Biological Chemistry*, *276*(16), 12645–12653. 10.1074/jbc.M011344200 11278891

[CIT0020] Yamauchi-Takihara, K., Ihara, Y., Ogata, A., Yoshizaki, K., Azuma, J., & Kishimoto, T. (1995). Hypoxic stress induces cardiac myocyte-derived interleukin-6. *Circulation*, *91*(5), 1520–1524. 10.1161/01.CIR.91.5.1520 7867193

[CIT0021] Yanagisawa, M., Kurihara, H., Kimura, S., Tomobe, Y., Kobayashi, M., Mitsui, Y., Yazaki, Y., Goto, K., & Masaki, T. (1988). A novel potent vasoconstrictor peptide produced by vascular endothelial cells. *Nature*, *332*(6163), 411–415. 10.1038/332411a0 2451132

